# Long-term follow-up and pregnancy after complete sacrectomy with lumbopelvic reconstruction: case report and literature review

**DOI:** 10.1186/s12884-015-0735-5

**Published:** 2016-01-04

**Authors:** Valentin V. Barsan, Valentina Briceño, Manisha Gandhi, Andrew Jea

**Affiliations:** Neuro-Spine Program, Division of Pediatric Neurosurgery, Department of Neurosurgery, Baylor College of Medicine, Texas Children’s Hospital, 6621 Fannin Street, CCC 1230.01, 12th Floor, Houston, TX 77030 USA; Department of Obstetrics and Gynecology, Baylor College of Medicine, Texas Children’s Hospital, 6621 Fannin Street, CCC 1230.01, 12th Floor, Houston, TX 77030 USA

**Keywords:** HRQoL outcomes, Myofibroblastic sarcoma, Pediatric spine, Pregnancy, Obstetrics, Sacrectomy

## Abstract

**Background:**

Sacrectomy remains a technically complex procedure for resection of malignant pelvic neoplasia. Commonly, postoperative complications include permanent neurological deficits. Only a few studies have reported the long-term functional outcomes of patients who had undergone sacrectomy.

**Case presentation:**

We previously reported on the utilization of complete sacrectomy and lumbopelvic reconstruction for the management of primary myofibroblastic sarcoma of the sacrum and ilium in a 15-year-old female patient. In this report, we update her postoperative course with an additional 5 years of follow-up and Health-Related Quality of Life (HRQoL) outcomes. During this time period, she gave birth to two healthy full-term babies.

**Conclusion:**

To the best of our knowledge, this is the first report of pregnancy after total sacrectomy and lumbopelvic reconstruction. We outline some of the challenges in the obstetrical management of this patient.

## Background

The risk of neurological morbidity following sacrectomy is high. Several reports have documented such long-term outcomes in patients who had undergone sacrectomy for primary sacral tumors [1–8]. In pediatric patients, however, long-term outcomes after sacrectomy for sacral tumors are rarely elucidated in the published literature.

We previously had published our 1-year experience with a 15-year-old female patient who had undergone a complete sacrectomy with lumbopelvic reconstruction for a low-grade myofibroblastic sarcoma of the sacrum. Now, 5 years after surgery, we update our previous report. Most remarkably, the patient has had two successful pregnancies resulting in the birth of two babies at term without perinatal complications. To our knowledge, pregnancy after sacrectomy has not been previously reported in the literature.

## Case presentation

### History and physical examination

Our patient was a 20-year-old woman with a history of a sacral mass diagnosed by CT-guided needle biopsy 7 years before as benign fibrohistiocytoma. A repeat biopsy of her lesion through an open approach revealed myofibroblastic sarcoma. Consideration was given for reduction of tumor burden and lumbopelvic reconstruction to facilitate long-term progression-free survival and to improve quality of life, respectively.

### Operation for complete sacrectomy and lumbopelvic reconstruction

The extension of tumor into both sacroiliac joints prohibited sparing S1; thus, a two-staged total sacrectomy was planned, as previously described. Briefly, the first stage of the procedure included a midline laparotomy, mobilization of the visceral and neural structures, and ligation of the internal iliac vessels. A colostomy was performed, and a right vertical rectus abdominus myocutaneous flap based on the inferior epigastric vessels was mobilized, wrapped in a bowel bag, and placed in the pelvis. The second stage was performed the next day, and it included L5 and S1 laminectomies, bilateral osteotomies and disarticulation of the sacrum from the ilium at the sacroiliac joints, ligation of the thecal sac inferior to the takeoff of the L5 nerve roots, complete L5–S1 discectomy, and transection of the S1–S5 nerve roots. The entire sacrum, along with the tumor, was removed piecemeal. This was followed by lumbopelvic reconstruction with spinal instrumentation and bone graft.

### Postoperative course

The patient underwent a long inpatient postoperative course. She responded well to intensive physical rehabilitation. Her postoperative course was complicated by the development of a small superficial soft tissue *Staphylococcus* epidermidis abscess in the operative bed treated with percutaneous drainage and a full course of intravenous and oral antibiotics. The patient also developed a small ischial decubitus ulcer, remote from the surgical incision, treated with local wound care. At 3 months after surgery, the patient was able to ambulate with the assistance of a walker. She had normal strength in her left leg, except for plantarflexion (S1). Her right leg was more impaired, with normal proximal strength but significant weakness in hip extension (L4 and L5), dorsiflexion (L4), extensor hallucis longus (L5), and plantarflexion (S1), suggesting sciatic nerve injury. She was discharged home and was pain-free off narcotics.

**Fig. 1:**
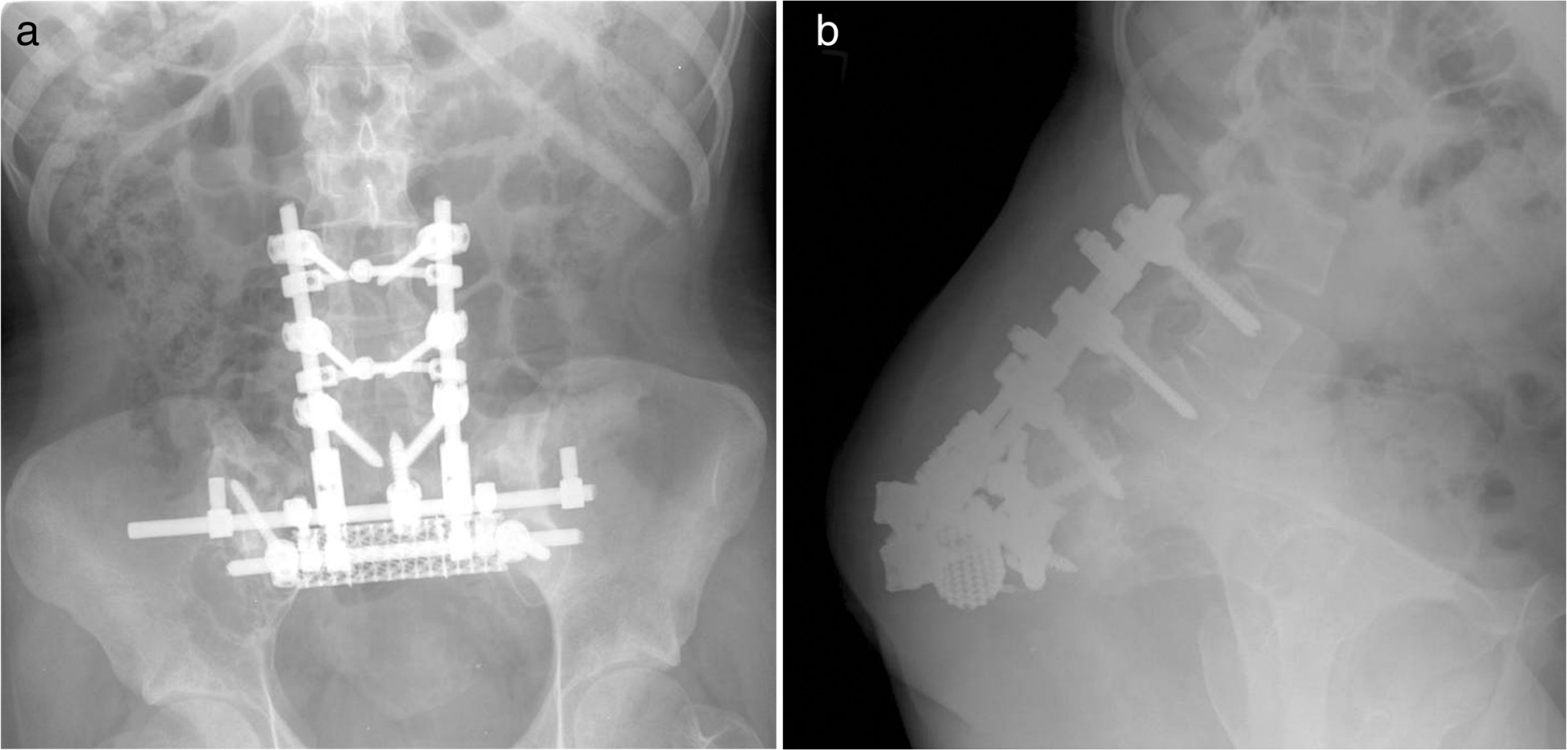
Upright (**a**) AP and (**b**) lateral X-rays show no evidence of instrumentation failure with maintenance of spinal and pelvic alignment at 5 years after sacrectomy

At 5 years after surgery, the patient remains pain-free. She was able to ambulate independently with a right ankle-foot orthosis. There was no evidence of locally progressive or metastatic disease on follow-up imaging. Postoperative imaging showed settling and a stable fibrous pseudoarthrosis (Fig. 1a and b).

### Pregnancies

The patient’s first pregnancy was unbeknownst to us during a period of follow-up loss after sacrectomy. However, she gave birth to a healthy 5-lb, 2-oz baby girl through a cesarean delivery at 37 weeks’ gestation by a community obstetrician. There were no birth complications. The baby, now 3½ years old, is meeting developmental milestones.

**Fig. 2:**
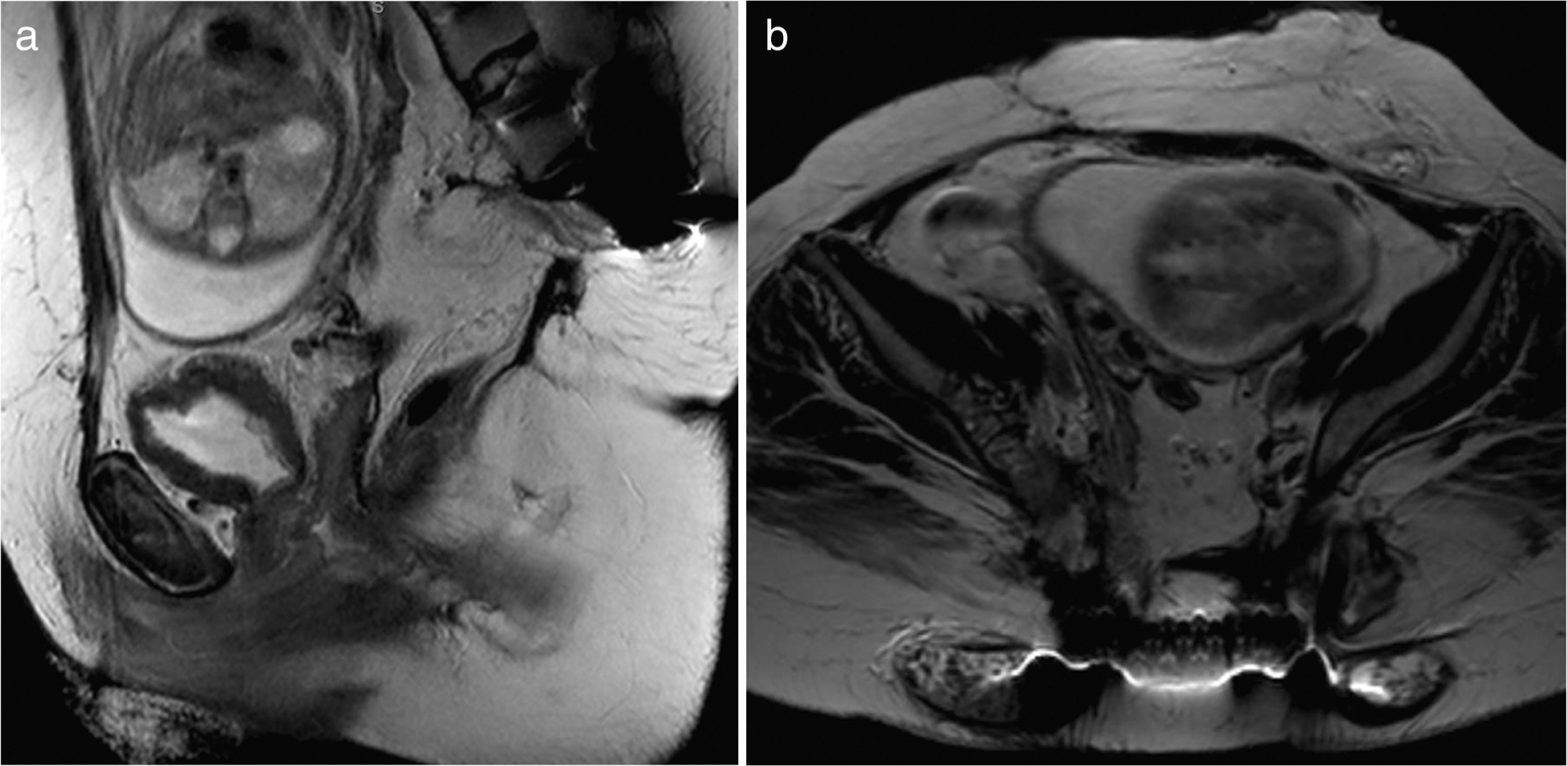
**a** Prenatal sagittal T2-weighted MR image demonstrates the uterus and fetus with their relationship to the lumbopelvic instrumentation (metal artifact). **b** Prenatal axial T2-weighted MR image with gadolinium shows non-progressive stable disease in the iliac wings

**Fig. 3:**
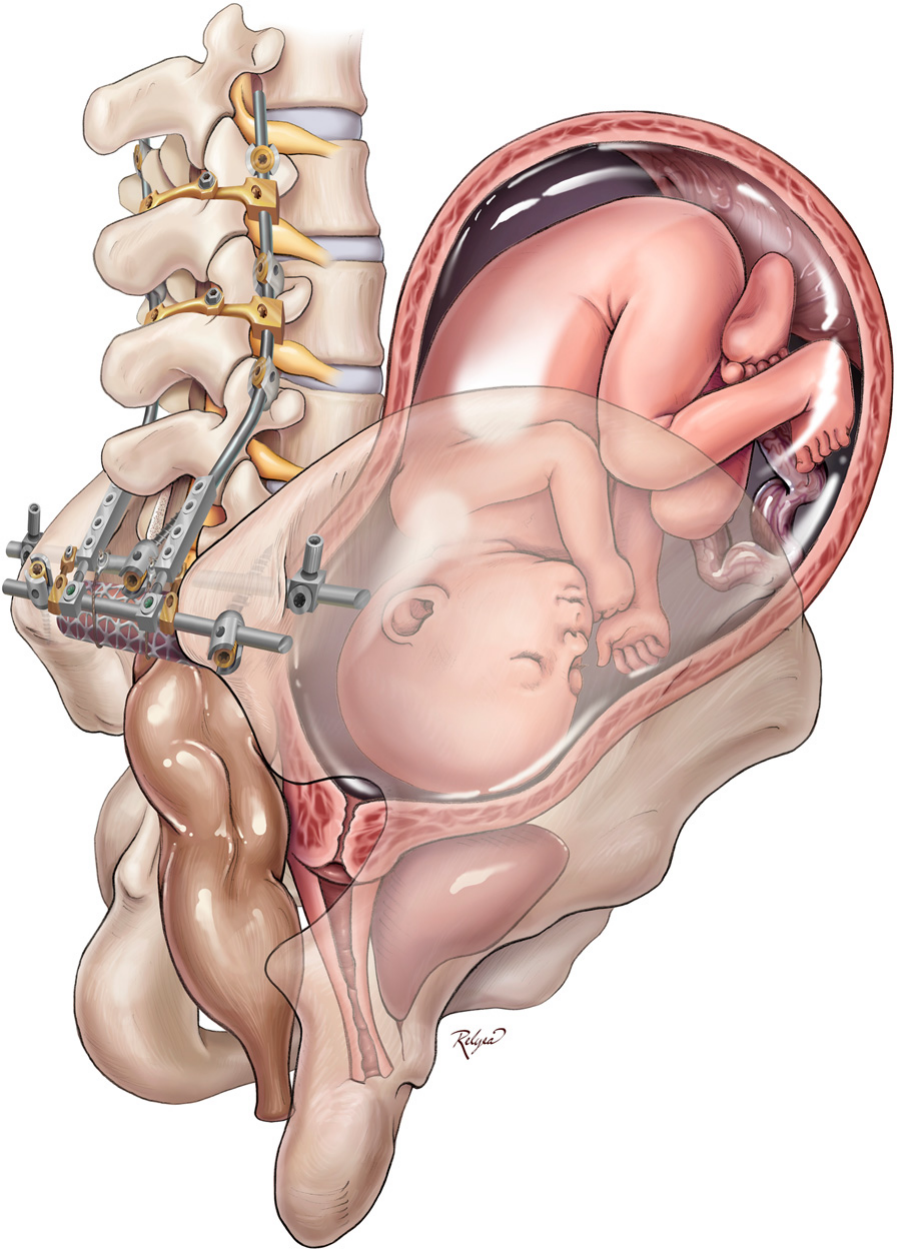
Artist’s illustration depicts the pregnant uterus in context with the lumbopelvic reconstruction after total sacrectomy

**Fig. 4:**
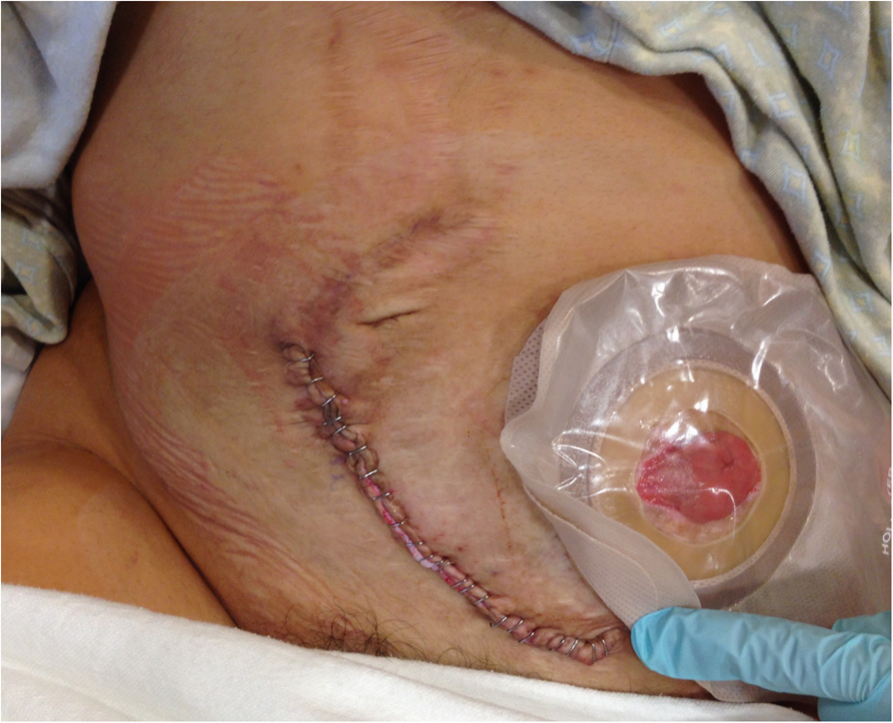
The cesarean incision follows along the previous midline laparotomy and rectus abdominis donor incision. Once the incision is opened, the inferior epigastric vessels, the vascular pedicle for the posterior myocutaneous flap, are identified and preserved

**Fig. 5:**
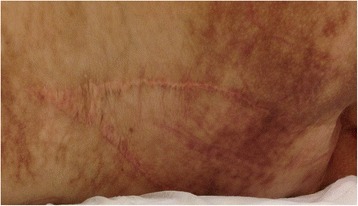
Photograph of the site of sacral defect repair, seen 5 years after surgery and immediately after cesarean, demonstrates viability of myocutaneous flap

**Fig. 6:**
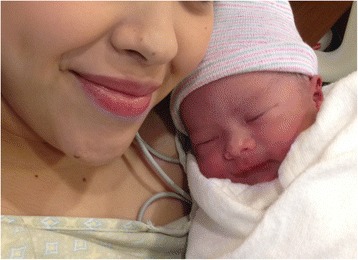
Photograph of the healthy second baby with our patient shortly after cesarean delivery

The Baylor Maternal-Fetal Medicine Service at Texas Children’s Pavilion for Women followed her second pregnancy closely. Frequent visits every 2 to 3 weeks were scheduled to monitor maternal status (e.g., weight gain and blood pressure). The pregnancy was carefully documented with monthly fetal ultrasound and MRI (Figs. 2a, b, and 3). At each outpatient visit, catheterization was performed to send urine culture, and the patient was on Keflex suppression throughout the pregnancy. Routine prenatal labs were obtained, and routine vaccines were administered. A repeat elective cesarean was performed at 37 weeks of gestation. Great care was taken to identify the right inferior epigastric vessels, the pedicle of the rectus abdominis musculocutaneous flap used for closure of the sacral defect (Figs. 4 and 5). Plastic surgery was available at the time of delivery, but the right rectus abdominis muscle flap was not seen and was assumed to be posterior to the uterus. A healthy baby girl was delivered (Fig. 6). The birth weight was recorded as 6 lb and 9 oz. Apgar scores of 8 and 9 were assigned at 1 and 5 min, respectively.

### Timeline of patient history

Table 1 summarizes the patient history in timeline format.

**Table 1:** Timeline of patient’s history

Age	13 years old	15 years old	16 years old	20 years old
			*Lost to follow-up*	
Events	Sacral mass diagnosed as benign fibrohistiocytoma by needle biopsy	Open biopsy of sacral mass reveals myofibroblastic sarcoma	Birth of first child	Birth of second child
		Failure of the tumor to respond to chemotherapy prompts attempt at aggressive resection		Pain-free and no progression of residual disease
		Complete sacrectomy with lumbopelvic reconstruction performed		Spinal instrumentation stable

### HRQoL outcomes

Prior to the patient’s second pregnancy, we collected standardized and validated indices data to quantitate Health-Related Quality of Life (HRQoL) outcomes through the Oswestry Disability Index (ODI) and MOS Short Form 36 (SF-36).

Our patient’s ODI score was 30 %, representing mild disability.

SF-36 Physical Component Summary for our patient was calculated as 28.6 (national average, 53.5), and SF-36 Mental Component Summary was 52.9 (national average, 46.19). Our patient’s scores compared favorably in the domains of social functioning and mental health but were below the population norms across the remaining scales of physical functioning.

## Conclusions

### Total sacrectomy

The treatment of primary sacral tumors represents a challenge because of their anatomical location and often large size at presentation. Instability at the lumbopelvic junction frequently accounts for the majority of the symptoms, such as axial back pain, that these patients, like ours, experience. Reconstruction of the lumbopelvic junction succeeds in stabilizing the spine from a biomechanical standpoint, which improves patient functionality. There have been no other reports archiving pregnancy after sacrectomy.

### Obstetrical considerations after sacrectomy

#### Neurologic

The neurological morbidity—skin breakdown, infections, deep venous thrombosis (DVT), autonomic dysreflexia, contractures, spasticity, neurogenic bladder and bowel, and progressive spinal deformity—observed after sacrectomy amounts to an iatrogenic “spinal injury.”

#### Genitourinary

Pyelonephritis and urinary tract infections are associated with higher risk of spontaneous prematurity. Because of the increased risk and severity of urinary tract infections, more frequent assessment for asymptomatic bacteriuria and antibiotic suppressive therapy has been suggested for pregnant women with neurogenic bladders.

#### Cutaneous

Pressure ulcer prevalence is significant throughout the lifetime of a person with impaired mobility. Pregnancy can be a risk factor for this complication due to further decreased mobility, development of dependent edema at the lower extremities, weight gain, and nutritional deficiencies. Prevention remains the cornerstone. Pregnancy-specific preventive recommendations include more frequent changes in body position, reassessment of equipment (wheelchair, cushion, and orthosis), and greater attention to limiting weight gain during pregnancy.

#### Pulmonary

Pregnancy presents multiple potential risk factors for thromboembolism that may act synergistically with neurological injury resulting in paraparesis. They include greater immobility, preeclampsia, sepsis, and cesarean delivery. Although we discussed risk for venous thromboembolism (VTE) due to decreased mobility, we did not take additional measures, such as prophylactic low-molecular-weight heparin, and simply encouraged ambulation. In general, however, thromboprophylaxis is indicated for cesareans in women with neurological deficits.

#### Musculoskeletal

A number of changes associated with pregnancy may result in a loss of independence. Worsening spasticity of abdominal or adductor muscles—in addition to weight gain and carpal tunnel syndrome frequently occurring during pregnancy, even in women without neurological deficits—may lead to difficulty in transferring, requiring the use of a wheelchair or utilization of a walker at the end of pregnancy in up to half of women with paraparesis.

#### Cesarean section versus vaginal delivery

Neurological deficit is not an absolute indication for cesarean delivery. Nonetheless, the rate of cesareans among women with neurological dysfunction is higher than expected in the general obstetric population. Our patient had a prior cesarean delivery, declined a trial of labor after cesarean, and elected for repeat cesarean section. Vaginal delivery could have been an option, but the concern was that in case of an emergency cesarean delivery, there could be surgical risks due to the extensive surgery that she had had before.

#### Consultation with plastic surgery

A vertical rectus abdominis myocutaneous flap has one of the smallest incidences of necrosis of any of the myocutaneous flaps associated with pelvic reconstructive surgery. The physiologic key is that an open wound has been covered with a myocutaneous flap that offers an excellent covering for a wound with an excellent blood supply, i.e. the inferior epigastric artery, a branch of the external iliac artery. Extreme care should be taken to ensure the integrity of the inferior epigastric vessels as they branch off the external iliac vessels. When the neurovascular bundle of the inferior epigastric artery has been interrupted, the flap likely will not survive. Thus, it would be wise to involve a plastic surgeon in the planning of a cesarean section incision.

### Effects on pregnancy outcomes

Women with neurological impairment may be insensate to the onset of labor. Instructions on techniques of self-uterine palpation to detect contractions at home and serial assessment of cervical length with ultrasonography and home uterine activity monitoring may constitute additional measures to prevent unexpected spontaneous prematurity or undiagnosed labor.

Postpartum depression has been reported to be higher in women with neurological deficits than in the general pregnant population. Correlates of depression include higher stress level, greater social isolation and lower satisfaction with social network, less mobility, unemployment, abuse, and poorer overall health.

### Summary

We treated a 15-year-old female patient who underwent a total sacrectomy for gross total resection (GTR) of a sacral myofibroblastic sarcoma with extension into the iliac wings and, herein, have described long-term (5-year) functional follow-up. Pregnancy after sacrectomy has not been previously reported in the literature. During the follow-up period, our patient carried two babies to term and delivered via cesarean with no perinatal complications. Obstetric challenges and considerations for our patient with neurological deficits in lower extremity, impaired mobility, and bladder and bowel dysfunction include the following: prophylaxis for urinary tract infections, DVT prevention, precautions against pressure ulcers, maintenance of functional independence, and preservation of the blood supply (i.e., the inferior epigastric artery) to the myocutaneous flap used during the pelvic reconstruction when planning a cesarean. Long-term progression-free survival with satisfactory HRQoL outcomes seems to have been attained.

### Consent statement

Written informed consent was obtained from the patient for publication of this case report and any accompanying images. A copy of the written consent is available for review.

## Abbreviations

CT: Computed tomography
DVT: Deep vein thrombosis
GTR: Gross total resection
HRQoL: Health-Related Quality of Life
ODI: Oswestry Disability Index
SF-36: MOS Short Form 36
VTE: Venous thromboembolism

## References

[1] Bergh P, Gunterberg B, Meis-Kindblom JM, Kindblom LG (2001). Prognostic factors and outcome of pelvic, sacral, and spinal chondrosarcomas: a center-based study of 69 cases. Cancer.

[2] Bethke KP, Neifeld JP, Lawrence W (1991). Diagnosis and management of sacrococcygeal chordoma. J Surg Oncol.

[3] Davidge KM, Eskicioglu C, Lipa J, Ferguson P, Swallow CJ, Wright FC (2010). Qualitative assessment of patient experiences following sacrectomy. J Surg Oncol.

[4] DeVivo MJ, Black KJ, Stover SL (1993). Causes of death during the first 12 years after spinal cord injury. Arch Phys Med Rehabil.

[5] Fernández-Aceñero MJ, Sanz-Laguna A, Carrascoso-Arran J, López-Criado P: Low-grade myofibroblastic sarcoma of the bone [http://ispub.com/IJPA/4/1/8428 Accessed 4 January 2016.]

[6] Fourney DR, Rhines LD, Hentschel SJ, Skibber JM, Wolinsky JP, Weber KL (2005). En bloc resection of primary sacral tumors: classification of surgical approaches and outcome. J Neurosurg Spine.

[7] Gottfried ON, Omeis I, Mehta VA, Solakoglu C, Gokaslan ZL, Wolinsky JP (2011). Sacral tumor resection and the impact on pelvic incidence. J Neurosurg Spine.

[8] Hulen CA, Temple HT, Fox WP, Sama AA, Green BA, Eismont FJ (2006). Oncologic and functional outcome following sacrectomy for sacral chordoma. J Bone Joint Surg Am.

